# Anti-Vivisection Statistics

**Published:** 1904-10-01

**Authors:** Robt. Stewart

**Affiliations:** Secretary


### EDITOR'S LETTER-BOX.

[Our Correspondents are reminded that prolixity is a great bar to publication, and that brevity of style and conciseness of statement greatly facilitate early insertion.]

### ANTI-VIVISECTION STATISTICS.

*To the Editor of* The Hospital.

September 26th, 1904.

Dear Sir,—Mr. Coleridge desires me to state, in reply to the inquiry made in a paragraph of your paper, dated September 24th, that the reason he left the Poplar Hospital out of the hospitals mentioned by him to the *Morning Post* and other London papers, was that he dealt only with general hospitals, and the Poplar Hospital is devoted entirely to accidents. Yours faithfully,

Robt. Stewart, Secretary.

